# Optimizing the use of ketamine to reduce chronic postsurgical pain in women undergoing mastectomy for oncologic indication: study protocol for the KALPAS multicenter randomized controlled trial

**DOI:** 10.1186/s13063-023-07884-y

**Published:** 2024-01-19

**Authors:** Jing Wang, Lisa V. Doan, Deborah Axelrod, John Rotrosen, Binhuan Wang, Hyung G. Park, Robert R. Edwards, Michele Curatolo, Carina Jackman, Raven Perez, NCATS Trial Innovation Network

**Affiliations:** 1 Department of Anesthesiology, Perioperative Care, and Pain Medicine, NYU Grossman School of Medicine, New York, NY USA; 2 Department of Neuroscience and Physiology, NYU Grossman School of Medicine, New York, NY USA; 3 Department of Surgery, NYU Grossman School of Medicine, New York, NY USA; 4 Department of Psychiatry, NYU Grossman School of Medicine, New York, NY USA; 5 Department of Population Health, NYU Grossman School of Medicine, New York, NY USA; 6 Department of Anesthesia, Brigham and Women’s Hospital, Boston, MA USA; 7 Department of Anesthesiology and Pain Medicine, University of Washington, Seattle, WA USA; 8 Department of Anesthesiology, University of Utah, Salt Lake City, UT USA

**Keywords:** Postoperative pain, Chronic postsurgical pain, Postmastectomy pain syndrome, Ketamine, Non-opioid

## Abstract

**Background:**

Mastectomies are commonly performed and strongly associated with chronic postsurgical pain (CPSP), more specifically termed postmastectomy pain syndrome (PMPS), with 25–60% of patients reporting pain 3 months after surgery. PMPS interferes with function, recovery, and compliance with adjuvant therapy. Importantly, it is associated with chronic opioid use, as a recent study showed that 1 in 10 patients continue to use opioids at least 3 months after curative surgery. The majority of PMPS patients are women, and, over the past 10 years, women have outpaced men in the rate of growth in opioid dependence. Standard perioperative multimodal analgesia is only modestly effective in prevention of CPSP. Thus, interventions to reduce CPSP and PMPS are urgently needed. Ketamine is well known to improve pain and reduce opioid use in the acute postoperative period. Additionally, ketamine has been shown to control mood in studies of anxiety and depression. By targeting acute pain and improving mood in the perioperative period, ketamine may be able to prevent the development of CPSP.

**Methods:**

Ketamine analgesia for long-lasting pain relief after surgery (KALPAS) is a phase 3, multicenter, randomized, placebo-controlled, double-blind trial to study the effectiveness of ketamine in reducing PMPS. The study compares continuous perioperative ketamine infusion vs single-dose ketamine in the postanesthesia care unit vs placebo for reducing PMPS. Participants are followed for 1 year after surgery. The primary outcome is pain at the surgical site at 3 months after the index surgery as assessed with the Brief Pain Inventory-short form pain severity subscale.

**Discussion:**

This project is part of the NIH Helping to End Addiction Long-term (HEAL) Initiative, a nationwide effort to address the opioid public health crisis. This study can substantially impact perioperative pain management and can contribute significantly to combatting the opioid epidemic.

**Trial registration:**

ClinicalTrials.gov NCT05037123. Registered on September 8, 2021.

**Supplementary Information:**

The online version contains supplementary material available at 10.1186/s13063-023-07884-y.

## Administrative information

Note: The numbers in curly brackets in this protocol refer to SPIRIT checklist item numbers. The order of the items has been modified to group similar items (see http://www.equator-network.org/reporting-guidelines/spirit-2013-statement-defining-standard-protocol-items-for-clinical-trials/).

**Table:** 

Title {1}	**Optimizing the use of ketamine to reduce chronic postsurgical pain in women undergoing mastectomy for oncologic indication: study protocol for the KALPAS multicenter randomized controlled trial**
Trial registration {2a and 2b}	ClinicalTrials.gov NCT05037123. This trial was registered on September 8, 2021
Protocol version {3}	The current approved protocol is version 3, approved February 23, 2022
Funding {4}	This research is supported by the National Institutes of Health through the NIH HEAL Initiative under award number UH3CA261067. Research reported in this publication is also supported by the NCATS Trial Innovation Network (TIN), under award numbers U24TR001608-05S3 (clinical coordinating center), U24TR001597-06S1 (data coordinating center), U24TR001579-06S1 (Recruitment Innovation Center), and U24TR001609 (Safety and Statistical Coordinating Center)
Author details {5a}	Jing Wang, MD, PhD, New York University Grossman School of Medicine Lisa V Doan, MD, New York University Grossman School of Medicine Deborah Axelrod, MD, New York University Grossman School of Medicine John Rotrosen, MD, New York University Grossman School of Medicine Binhuan Wang, PhD, New York University Grossman School of Medicine Hyung G Park, PhD, New York University Grossman School of Medicine Robert R Edwards, PhD, Brigham and Women’s Hospital Michele Curatolo, MD, PhD, University of Washington Carina Jackman, MD, University of Utah Raven Perez, BA, New York University Grossman School of Medicine
Name and contact information for the trial sponsor {5b}	National Cancer Institute (NCI) 9609 Medical Center Drive Building 9609 MSC 9760 Bethesda, MD 20892–9760
Role of sponsor {5c}	The content is solely the responsibility of the authors and does not necessarily represent the official views of the National Institutes of Health

## Introduction

### Background and rationale {6a}

Approximately, 20% of postoperative patients develop chronic postsurgical pain (CPSP), defined as pain related to surgery lasting greater than 3 months, making it one of the most common forms of chronic pain [1–3]. CPSP is associated with persistent opioid use and dependence [1–4]. Mastectomies are commonly performed in the USA and have a particularly strong association with CPSP, where 25–60% of patients continue to experience pain more than 3 months after surgery [5–12]. Chronic pain after mastectomy, termed postmastectomy pain syndrome (PMPS), may be caused initially by damage to peripheral nerves (e.g., intercostobrachial nerve) and/or tissues during surgery and maintained by maladaptive plasticity in the central nervous system [1–10]. PMPS interferes with function, recovery, and compliance with adjuvant therapy. Studies have identified specific risk factors for CPSP, including preoperative pain, severe pain after surgery, anxiety, depression, pain catastrophizing, and surgical factors [11–14]. Patients undergoing breast cancer surgery have particularly high levels of preoperative anxiety and depression, key risk factors for the development of CPSP [6, 8–13]. Standard perioperative multimodal analgesia using a combination of opioids, non-opioids, and in some cases regional anesthesia is only moderately effective in prevention of CPSP. Thus, interventions to reduce CPSP in general and PMPS in particular are urgently needed.

Ketamine has several important clinical properties supporting its use in CPSP. First, at subanesthetic doses, ketamine can dramatically reduce acute pain severity with mild side effects [15–18]. Second, as a dissociative analgesic, it can alter brain plasticity to dissociate the affective from the sensory component of pain to reduce pain aversion and pain catastrophizing [19–21]. Lastly, by modifying brain plasticity, ketamine, given at single doses of 0.3–0.6 mg/kg, has antidepressant effects lasting several weeks, and, thus, it is used as a bridge therapy for depression [22–25]. Acute pain severity, pain catastrophizing, depression, and anxiety are all major risk factors for chronic pain; hence, its acute analgesic and dissociative properties and long-lasting mood-elevating effects make ketamine a promising agent for the prevention of CPSP, which has long been an elusive and challenging goal [26].

Numerous studies, including studies from our group, have shown that continuous perioperative low-dose ketamine infusion relieves postsurgical pain, reduces opioid use [27–49], and improves function [27, 29, 30]. A number of studies showed that ketamine can also reduce the severity of CPSP [27, 50–52]. In these studies, however, dosing regimens for ketamine varied widely, and study populations were heterogeneous. Thus, large multisite studies with standardized treatment regimens are needed to establish the efficacy of ketamine for prevention of CPSP. In addition, prior studies of ketamine for postsurgical pain typically use continuous infusions of ketamine, variably during surgery and/or the postoperative period. As an alternative, a single-dose of ketamine (0.3–0.6 mg/kg) can effectively activate the cortical top-down system for mood regulation [19, 20, 53–64] and has been used in the emergency department to provide long-lasting post-discharge pain relief and minimize opioid prescriptions [65–67]. In a recent pilot randomized controlled trial (RCT) of single-dose ketamine in the postanesthesia care unit (PACU), we found that ketamine reduced pain for 7 days after bariatric surgery [68]. Requirements for monitoring patients during continuous ketamine infusion vary by hospital, with some requiring intensive care level of monitoring. If found to be as effective as continuous ketamine infusion, a single dose of ketamine would be a highly practical and scalable treatment option that could be used in a variety of practice settings.

### Objectives {7}

The primary objective is to determine the effectiveness of continuous ketamine infusion and single-dose ketamine to reduce pain at the surgical site at 3 months after surgery as assessed by the Brief Pain Inventory (BPI) pain severity subscale. Secondary outcomes include pain severity and interference at the surgical site, incidence of PMPS, anxiety, and depression over 12 months after surgery. Tertiary outcomes include assessment of neuropathic symptoms, fatigue, sleep, physical function, and opioid use.

### Trial design {8}

This is a multicenter, three arm, double-blind, RCT to test the effectiveness of continuous ketamine infusion or single dose of ketamine to reduce PMPS in women undergoing mastectomy for oncologic indication. The primary efficacy analysis examines differences in pain at 3 months between the continuous ketamine infusion and the control or between single-dose ketamine and control. As a secondary hypothesis for the primary endpoint, non-inferiority of the single-dose ketamine arm to the continuous ketamine infusion arm will be tested.

## Methods: participants, interventions and outcomes

### Study setting {9}

Ketamine analgesia for long-lasting pain relief after surgery (KALPAS) is a phase 3, multicenter, randomized, placebo-controlled, double-blind trial to study the effectiveness of ketamine in reducing PMPS. It will be conducted in accordance with the International Council for Harmonisation (ICH), E6: Good Clinical Practice guidelines (GCP). The study was approved by the University of Utah Institutional Review Board and registered on ClinicalTrials.gov NCT05037123. Enrolling sites include NYU Langone Health, University of Washington Medical Center, Brigham and Women’s Hospital, Mayo Clinic, MD Anderson Cancer Center, Memorial Sloan Kettering Cancer Center, Montefiore Einstein, New York Presbyterian Columbia University Irving Medical Center, Rush University Medical Center, University of Alabama at Birmingham, University of Arkansas for Medical Sciences, University of Pittsburgh Magee Women’s Hospital, University of Texas Southwestern Medical Center, Washington University Medical Center, MetroHealth Medical Center, and University of Cincinnati Medical Center.

### Eligibility criteria {10}

Participants’ inclusion criteria are as follows: women 18 years of age or older; undergoing elective breast surgery for oncologic indication such as unilateral or bilateral mastectomy, prophylactic mastectomy, + / − lymph node dissection, and + / − immediate or delayed reconstruction; and no distant metastases.

An individual who meets any of the following criteria will be excluded from participation in this study: (1) history of cognitive impairment or clinical signs of altered mental status (AMS) that may interfere with adherence to study procedures and/or participant safety (clinical signs of AMS may include but are not limited to confusion, amnesia, disorientation, fluctuating levels of alertness, etc.); (2) past ketamine or phencyclidine misuse or abuse; (3) schizophrenia or history of psychosis; (4) history of post-traumatic stress disorder; (5) known sensitivity or allergy to ketamine; (6) liver or renal insufficiency; (7) history of uncontrolled hypertension, chest pain, cardiac arrhythmia, stroke, head trauma, intracranial mass or hemorrhage, glaucoma, porphyria, uncontrolled thyroid disease, or other contraindication to ketamine; (8) lamotrigine, alfentanil, physostigmine, or 4-aminopyridine use; (9) currently pregnant; (10) body mass index (BMI) greater than 41; (11) non-English or non-Spanish speaker; (12) currently participating in another pain interventional trial; (13) unwilling to comply with all study procedures and be available for the duration of the study; (14) patient is American Society of Anesthesiologists (ASA) physical status 4, 5, or 6; (15) patient has started or undergone hormone therapy for gender transition into male; or (16) patient is scheduled for bilateral (or greater) flap reconstruction.

### Who will take informed consent? {26a}

Informed consent is obtained for all study participants by study personnel. Consent can occur either in person or remotely via telephone or videoconferencing. Documented informed consent is done electronically.

### Additional consent provisions for collection and use of participant data and biological specimens {26b}

Participants consent to data storage and sharing by the NIH or data center selected by the NIH for future research use.

## Interventions

### Explanation for the choice of comparators {6b}

When ketamine is used in the perioperative period, it is typical for it to be administered as a continuous low-dose infusion [27–49]. An alternative is a single dose of ketamine. In a pilot RCT of single-dose ketamine in the PACU, ketamine reduced pain for 7 days after bariatric surgery [68]. If found to be as effective as continuous ketamine infusion, a single dose of ketamine would be a highly practical and scalable treatment option that could be used in a variety of practice settings.

### Intervention description {11a}

Prior to study intervention, participants will be randomly assigned to one of three arms, utilizing a parallel group (1:1:1 ratio) randomization design to receive continuous perioperative ketamine infusion vs. postoperative single-dose ketamine vs. matching placebo (saline). The assignments will be generated through the REDCap randomization module, or in case of emergency from a backup randomization envelope, which will provide a participant-specific randomization code that will be stored REDCap. Randomization will be stratified by site. Participants will be randomized with random size permuted blocks, blinded to investigators to prevent bias, to ensure balance in treatment arms throughout the study. Participants, surgeons, clinicians, and assessors will be blinded with respect to treatment assignments.

For participants in the continuous ketamine infusion arm, ketamine will be administered after anesthetic induction as a 0.35 mg/kg bolus followed by a 0.25 mg/kg/h infusion during surgery with a maximum infusion duration of 6 h intraoperatively. The study drug may be paused approximately 15 min prior to expected extubation in cases of general anesthesia at discretion of anesthesiologist for emergence. The infusion will be restarted for two additional hours in the PACU. To maintain blinding, a saline dose will be given in the PACU over approximately 50–60 min to mimic the single-dose arm. The PACU preparations are given simultaneously.

Participants in the single-dose ketamine arm will receive a single-dose of 0.6 mg/kg of ketamine in the PACU over approximately 50–60 min. To maintain blinding, participants will receive a dose of saline after induction, followed by a saline infusion intraoperatively and for 2 h in the PACU.

The placebo group will receive an intraoperative bolus of saline, followed by saline infusion intraoperatively, and for 2 h in the PACU. A single dose of saline will be given in the PACU over approximately 50–60 min.

### Criteria for discontinuing or modifying allocated interventions {11b}

Criteria for discontinuing the study intervention includes the following: allergic reaction thought to be related to study drug; uncontrolled severe hypertension, hypotension, or arrhythmia thought to be related to study drug; severe uncontrolled psychotomimetic side effects; or severe respiratory event in the PACU, such as reintubation, laryngospasm, or bronchospasm. Side effects will be monitored by clinicians caring for patients and can be treated pharmacologically or, in the event of some psychotomimetic side effects, with reassurance. If study drug is halted, it may be restarted depending on stability of participant and clinical scenario.

### Strategies to improve adherence to interventions {11c}

Enrolling sites complete a series of study training sessions as part of the site activation process. Clinical site monitoring visits are performed to ensure the study is implemented in accordance with the protocol.

### Relevant concomitant care permitted or prohibited during the trial {11d}

Ketamine use will be restricted to study intervention. Otherwise, as a pragmatic trial in which ketamine is being studied as an adjunctive and preventive treatment, there are no other restrictions on anesthetic medications or techniques as well as postoperative analgesics or complementary and alternative therapies.

### Provisions for posttrial care {30}

There is no posttrial care.

### Outcomes {12}

The primary outcome is pain at the surgical site at 3 months after index surgery as assessed with the Brief Pain Inventory-short form (BPI) pain severity subscale. BPI is recommended for use in clinical trials for acute and chronic pain [69, 70]. The pain severity subscale is the mean of four items measuring current pain, pain on average, and pain at its worst and least in the past 24 h) on a scale from 0 (no pain) to 10 (worst pain). BPI has good validity and reliability in therapeutic studies [71, 72]. Secondary and tertiary outcomes are presented in Table 1.

**Table 1:** Secondary and tertiary outcomes

Objective	Brief description/justification of outcome measure	Outcome measured by	Time frame
**Pain outcomes *(secondary)***			
To determine the effectiveness of continuous ketamine infusion and single-dose ketamine to reduce pain at the surgical site at 3 months compared to placebo	The Brief Pain Inventory short form (BPI) pain severity subscale assesses pain at its worst, least, average, and current in the past 24 h The average and worst scores range from 0 to 10 for each item. These two separate items will be used	BPI average and worst pain of the severity subscales	3 months after surgery
To determine the effectiveness of single-dose vs continuous ketamine vs placebo on reducing pain severity and pain interference at multiple time points within 12 months after surgery	BPI assesses pain severity and interference. The interference subscale measures how much pain has interfered with general activities such as walking and working. Additionally, it inquires about interference in mood, enjoyment of life, relationships, and sleep	BPI pain severity and pain interference subscales	Baseline, 1 and 7 days, and 1, 3, 6, and 12 months after surgery
To determine the effectiveness of single-dose vs continuous ketamine vs placebo on reducing pain severity within 12 months after surgery	The Brief Pain Inventory short form (BPI) pain severity subscale assesses pain at its worst, least, average, and current in the past 24 h The average and worst scores range from 0 to 10 for each item. These two separate items will be used	BPI average and worst pain of the severity subscales	Baseline, 1 and 7 days, and 1, 3, 6, and 12 months after surgery
To determine the effectiveness of single-dose vs continuous ketamine vs placebo on reducing the incidence of PMPS	The BPI pain severity subscale assesses pain at its worst, least, average, and current in the past 24 h We will use participant’s average pain item to assess incidence of PMPS A score greater than 3 (0–10 scale) will be considered clinically meaningful chronic pain	BPI average pain score	Baseline, 3, 6, and 12 months after surgery
To determine the effectiveness of single-dose vs continuous ketamine vs placebo on pain in the surgical site (chest wall, axilla, and/or arm)	The Breast Cancer Pain Questionnaire (BCPQ) assesses pain location, frequency, and severity as well as sensory disturbance after breast surgery	BCPQ	7 days and 1, 3, 6, and 12 months after surgery
**Mood outcomes *(secondary)***			
To determine the effect of single-dose vs continuous ketamine vs placebo on *fatigue*	PROMIS fatigue is a questionnaire that assesses symptoms and feelings of tiredness *Fatigue is an important symptom in postoperative recovery*	PROMIS fatigue	Baseline, 7 days, and 1, 3, 6, and 12 months after surgery
**Pain outcomes *(tertiary)***			
To determine the effectiveness of single-dose vs continuous ketamine vs placebo on *reducing pain at multiple time points after receiving treatment*	The Patient Global Impression of Change (PGIC) is a part of the HEAL Core Data elements and it asks participants to report on changes in their pain levels compared to time of surgery, after receiving study intervention	PGIC	1, 3, 6, 12 months
To determine the effect of single-dose vs continuous ketamine vs placebo on *neuropathic symptoms*	PROMIS Neuropathic Scale is a questionnaire that assesses the incidence of neuropathic related pain symptoms PMPS may have a neuropathic component	PROMIS Neuropathic Scale	7 days and 1, 3, 6, and 12 months after surgery
**Function outcomes *(tertiary)***			
To determine the effect of single-dose vs continuous ketamine vs placebo on *fatigue*	PROMIS fatigue is a questionnaire that assesses symptoms and feelings of tiredness *Fatigue is an important symptom in postoperative recovery*	PROMIS fatigue	Baseline, 7 days and 1, 3, 6, and 12 months after surgery
To determine the effect of single-dose vs continuous ketamine vs placebo on *sleep quality and duration*	PROMIS sleep disturbance is a questionnaire that assesses sleep quality. Additionally, sleep duration will also be examined Sleep is an important symptom in postoperative recovery	PROMIS sleep disturbance, sleep duration	Baseline, 7 days and 1, 3, 6, and 12 months after surgery (Sleep duration at Baseline, 1, 3, 6, and 12 months)
To determine the effect of single-dose vs continuous ketamine vs placebo on *physical function*	PROMIS physical function is a questionnaire that assesses interference in physical function PMPS may impact physical functioning	PROMIS physical function	Baseline, 7 days, and 1, 3, 6, and 12 months after surgery
**Mood outcomes *(tertiary)***			
To determine the effect of single-dose vs continuous ketamine vs placebo on *fatigue*	PROMIS fatigue is a questionnaire that assesses symptoms and feelings of tiredness *Fatigue is an important symptom in postoperative recovery*	PROMIS fatigue	Baseline, 7 days, and 1, 3, 6, and 12 months after surgery

### Participant timeline {13}

The schedule of activities is presented in Table 2.

**Table 2:** Schedule of enrollment, intervention, and assessments

**Procedures**	Study visit 1 Screening and enrollment	Study visit 2 **Day of surgery (POD 0)** (perioperative)	Study visit 3 **POD 1** (+ 2 days)	Study visit 4 **POD 7** (+ 7 days)	Study visit 5 **1 month *(30 days)*** (− 3 days/ + 14 days)	Study visit 6 **3 months** (− 7 days/ + 14 days)	Study visit 7 **6 months** (− 7 days/ + 14 days)	Final study visit 8 **12 months** (− 7 days/ + 14 days)
Informed consent	X							
Review of inclusion/exclusion criteria	X							
Demographics	X							
Medical history and medication history	X							
TAPS — *Part 1 (part 2 only completed if participant’s scores a positive result from part 1)*	X						X	
Study intervention ketamine infusion or ketamine single dose or placebo		X						
***Pain questionnaires***								
Brief Pain Inventory (BPI) (assessing surgical site pain)	X		X	X	X	X	X	X
PROMIS-neuropathic pain quality 5a				X	X	X	X	X
Breast Cancer Pain Questionnaire (BCPQ)				X	X	X	X	X
Patient Global Impression of Change (PGIC)					X	X	X	X
***Mood questionnaires***								
PROMIS anxiety short form 4a	X			X	X	X	X	X
PROMIS depression short form 4a	X			X	X	X	X	X
Pain catastrophizing scale (PCS)	X					X		
Patient Health Questionnaire (PHQ)-2	X					X		
Generalized anxiety disorder (GAD)-2	X					X		
***Function questionnaires***								
PROMIS sleep disturbance-short form 6a	X			X	X	X	X	X
Sleep duration	X				X	X	X	X
PROMIS fatigue-short form 7b daily	X			X	X	X	X	X
PROMIS-physical function-short form 6b	X			X	X	X	X	X
**Analgesic use**								
Patient-reported analgesics	X	X	X	X	X	X	X	X
Inpatient analgesics (from medical records) *(Only complete if participants is still in the hospital)*		X	X	X	X	X	X	X
**Safety monitoring**								
Psycho-behavioral/side effects questionnaire (solicited AEs and SAEs)		X	X	X				
Assessment of adverse events and serious adverse events and side effects (unsolicited AEs and SAEs)		X	X	X				
Study discharge								X

### Sample size {14}

The sample size was determined based on the primary efficacy analysis. We conceptualized the study inference as having two pairwise comparisons: (1) the effect of continuous ketamine infusion vs. placebo and (2) the effect of single-dose ketamine vs. placebo, evaluated with respect to the primary outcome. The Bonferroni correction method was used to protect a familywise error rate (FWER) at 0.05, requiring each comparison to have a type-1 error rate of 0.025. In our sample size determination, to guard against potential skewed data distributions, we used nonparametric Wilcoxon’s rank-sum test. To detect an effect size of 0.30 (which corresponds to a hypothesized effect size of the single-dose treatment vs. placebo), 224 patients were required for each arm. In Table 3, to demonstrate the rationale of the sample size determination, we report the required sample size under various outcome distributions (and a different effect size), using both Wilcoxon’s rank-sum test and two-sample *t*-test. In total, to ensure 672 evaluable patients, we will recruit 750 patients, anticipating a 90% retention rate at 3 months.

**Table 3:** Minimal sample size required for an effect size (0.3 or 0.4) of ketamine treatment vs. control under various outcome distributions

Evaluable sample size per arm		Effect size			
		0.3 (e.g., 3.4 vs 4, *SD* = 2)		0.4 (e.g., 3.2 vs 4, *SD* = 2)	
		*t*-test	Wilcoxon’s test	*t*-test	Wilcoxon’s test
**Outcome distribution**	Normal	218	**224**	123	127
	Beta scaled to (0.10)	216	216	120	116
	Log-normal	221	132	122	74
	Logistic	213	186	121	104
	Laplace	216	139	122	83

The proposed study, however, is underpowered with respect to the non-inferiority test for single-dose ketamine vs. continuous ketamine infusion. With the non-inferiority margin chosen to be an effect size of 0.15 (which corresponds to the half of the hypothesized effect size, 0.3, of single-dose ketamine vs. placebo) and given 224 evaluable patients per each arm, we can achieve 46% power to detect non-inferiority using a one-sided Wilcoxon rank-sum test at the alpha level 0.05, when the actual mean difference between the single-dose arm and the continuous ketamine infusion arm is zero and the outcomes are normally distributed. Due to its low power with respect to the non-inferiority test, we will interpret the non-inferiority test results with much caution.

### Recruitment {15}

Recruitment strategies include printed recruitment materials, including brochures and posters that are placed in offices of collaborating surgeons. Digital recruitment materials include the study website and study informational video. Prescreening methods include clinician referrals, electronic health record system reports, and surgical schedules. Site staff reach out to potential participants to gauge interest and confirm study eligibility.

## Assignment of interventions: allocation

### Sequence generation {16a}

Prior to study intervention, participants will be randomly assigned to one of three arms, utilizing a parallel group (1:1:1 ratio) randomization design to receive continuous perioperative ketamine infusion vs. postoperative single-dose ketamine vs. matching placebo (saline). Randomization will be stratified by site. Participants will be randomized with random size permuted blocks.

### Concealment mechanism {16b}

The assignments will be generated through the REDCap randomization module, or in case of emergency from a backup randomization envelope, which will provide a participant-specific randomization code that will be stored in REDCap. The study drug will be prepared in a way that will not be visually distinguishable.

### Implementation {16c}

The randomization occurs no more than one business day prior to surgery. The randomization assignment is sent to the investigational pharmacy or designated personnel.

## Assignment of interventions: blinding

### Who will be blinded {17a}

Participants, surgeons, clinicians, and assessors will be blinded with respect to treatment assignments.

### Procedure for unblinding if needed {17b}

Criteria for discontinuing study intervention include reactions thought to be due to study drug, such as allergic reaction or uncontrolled, sustained severe hypertension. In rare instances, unblinding may be necessary such as during medical emergency where knowing whether ketamine was administered is critically necessary to provide appropriate care. In such situations, the unblinding request is first reviewed by the site principal investigator (PI). If the site PI believes the situation warrants unblinding, they will reach out to study PIs or study medical monitor to confirm unblinding decision. If the study PIs and medical monitor are unavailable, the site PI can move forward with their decision. If the request is approved, the unblended pharmacist will provide the group assignment.

## Data collection and management

### Plans for assessment and collection of outcomes {18a}

After informed consent, participants will complete a series of baseline assessments via REDCap. Data collection will take place through medical record abstraction and participant completion of assessments. Patient-reported outcomes will be assessed on postoperative days 1 and 7 and 1, 3, 6, and 12 months after surgery; these assessments may be done electronically, via telephone, or via paper forms, depending on participant preference. The study team will contact participants via phone or email in order to collect any missing data in an assessment during the specific timeframes for each visit.

### Plans to promote participant retention and complete follow-up {18b}

All participants will be asked to indicate their preferred mode of contact and provide multiple phone numbers and alternative contacts. REDCap will send automated reminders to participant’s emails and/or phones to prompt completion of follow-ups. The study team will make every attempt to collect assessments from participants. Participants may complete future assessments even if they have been unresponsive to previous assessments.

### Data management {19}

The University of Utah serves as the data coordinating center (DCC) and oversees data management. The DCC created the electronic data capture system in REDCap and is responsible for the security of the information system. Study data is largely generated by participant completion of self-reported assessments. Site research staff will use a combination of manual review, REDCap alerts, and automated queries to screen each completed survey for items that were not completed. Details of data management procedures can be found in the Manual of Operating Procedures.

### Confidentiality {27}

Participant confidentiality is strictly held in trust by the investigators, study staff, and study sponsor(s) and their agents. This confidentiality is extended to cover any study information collected relating to the participant, such as demographic information, medical history, and responses to assessments.

The study protocol, documentation, data, and all other information generated will be held in strict confidence. No information concerning the study or the data will be released to any unauthorized third party without prior written approval of the study sponsor. Additionally, the participants’ addresses collected at baseline to process payments or mail study documents will be deleted from the accessible database at the end of the study.

To further protect the privacy of study participants, the US Department of Health and Human Services (HHS) has issued a Certificate of Confidentiality to all researchers engaged in biomedical, behavioral, clinical, or other human subjects research funded wholly or in part by the federal government. As an NIH-funded study, this study protects identifiable research information from forced disclosure per the terms of the NIH policy. It is the NIH policy that investigators and others who have access to research records will not disclose identifying information except when the participant consents or in certain instances when federal, state, or local law or regulation requires disclosure.

### Plans for collection, laboratory evaluation, and storage of biological specimens for genetic or molecular analysis in this trial/future use {33}

This trial will not involve the collection of biological specimens.

## Statistical methods

### Statistical methods for primary and secondary outcomes {20a}

We will test the statistical significance of the differences in the primary outcome (the mean BPI pain severity subscale at 3 months) between (1) the continuous ketamine infusion and placebo and (2) the single-dose ketamine and placebo, each at 0.025 significance level (adjusted for multiple comparisons using the Bonferroni correction), based on two-sample *t*-tests (allowing unequal variances) if outcome variables are approximately normal or Wilcoxon’s rank-sum tests otherwise. The primary efficacy analysis will be performed on the modified intention-to-treat (mITT) sample, defined as those randomized and exposed to the study intervention, with analysis based on the randomly assigned treatment group.

In addition, we will use a linear mixed-effects model to quantify the effects of each treatment, adjusted for the pre-specified set of baseline (pre-randomization) covariates. The model will include study site (as a random intercept), two treatment indicators (continuous infusion vs. placebo and single dose vs. placebo), and the pre-specified set of baseline covariates. This set includes history of chronic pain (current vs. past or none), reconstruction (indicator variable: yes/no), axillary lymph node dissection (indicator variable: yes/no), chemotherapy (indicator variable: yes/no), immunotherapy (indicator variable: yes/no), and radiation (indicator variable: yes/no). An additional set of adjustment covariates will include patient baseline characteristics that are differentially distributed between treatment arms and deemed clinically significant. We will report the model-based effect estimate for each active treatment (continuous infusion and single dose) compared to the control with the associated 97.5% (adjusted for multiple comparisons using the Bonferroni correction) two-sided confidence intervals.

**Fig. 1:**
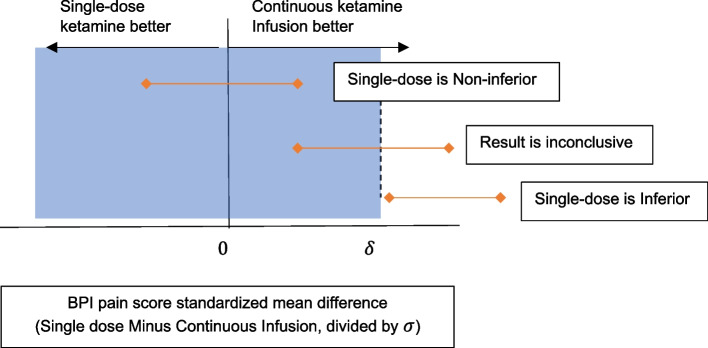
Non-inferiority testing for single-dose vs continuous ketamine infusion based on 90% confidence interval (in red) for the standardized difference in mean BPI severity at 3 months between single-dose and continuous infusion ketamine (adapted from Piaggio et al., 2012)

We will also conduct a non-inferiority test for comparing the single-dose ketamine arm to the continuous ketamine infusion arm at 0.05 one-sided level of statistical significance. We will set the non-inferiority margin $\delta$ to be an effect size of 0.15, which is half of the hypothesized effect size of 0.3 between the single-dose ketamine and placebo arms. Effect size is defined as the standardized difference in means between the two arms. For non-inferiority analysis, we will set the null hypothesis (H_0_) to be H_0_: $\frac{{\mu }_{single}- {\mu }_{infusion}}{\sigma }\ge \delta$ (i.e., the single-dose ketamine is inferior to the continuous ketamine infusion), whereas we will set the alternative hypothesis (H_a_) to be H_a_: $\frac{{\mu }_{single}- {\mu }_{infusion}}{\sigma }<\delta$ (i.e., the single-dose ketamine is as effective as the continuous ketamine infusion), where ${\mu }_{single}$ is the mean outcome for single-dose ketamine,${\mu }_{infusion}$ is the mean outcome for continuous ketamine infusion,$\sigma$ is the combined standard deviation (SD) of the outcome, and $\delta$ is the non-inferiority margin (set to be 0.15). Figure 1 shows the decision rule to conclude non-inferiority [73].

We will compute a 90% confidence interval on the effect size of single-dose vs continuous ketamine infusion using bias-corrected and accelerated (BCa) empirical bootstrap method. If the upper bound of the 90% confidence interval is less than $\delta$, then the null hypothesis (single-dose ketamine is inferior to continuous ketamine infusion) will be rejected, and the non-inferiority can be claimed (Fig. 1) at 0.05 one-sided level of statistical significance.

In addition to using the mean BPI pain severity subscale at 3 months, we will also perform similar analyses using the average and worst pain items in the BPI pain severity subscale at 3 months (for each item separately).

The secondary outcomes are postoperative pain and mood appraised at multiple time points within 12 months after surgery. The secondary outcomes include the mean BPI pain severity subscale, the BPI pain interference subscale, and the average and worst pain items in the BPI pain severity subscale, each assessed at baseline, 1 and 7 days, and 1, 3, 6, and 12 months after surgery; the incidence of PMPS (defined as the BPI average pain severity > 3) appraised at baseline, 3, 6, and 12 months after surgery; BCPQ assessed at 7 days and 1, 3, 6, and 12 months after surgery; and PROMIS Depression and Anxiety short form 4a, each appraised at baseline, 7 days and 1, 3, 6, and 12 months after surgery. These secondary outcome measures (obtained on more than one occasion) will be analyzed with a mixed-model repeated measures approach (MMRM). The basic MMRM model will include preoperative values of the outcome variable, treatment, factors for time, and treatment-by-time interaction, as well as random intercepts for study site and person. The pre-specified baseline variables (see the pre-specified variables in the primary analysis) and other variables that show imbalance between treatment arms will be included. In the MMRM, we will first use an unstructured covariance matrix for the residual covariance; however, if the model estimation fails to converge, we will choose the form of the residual covariance matrix, based on the Akaike’s information criterion, from candidate covariance structures including the auto-regressive-1 (AR1) and compound symmetry structures. The model covariance parameters will be estimated by restricted maximum likelihood (REML). Model-based treatment effect estimates (and 95% confidence intervals) for each time point will be used to present the effects of each treatment (the continuous ketamine infusion and the single-dose ketamine). Model-based estimates of the differences from the baseline (change from the pre-operative assessment) to each time point for each treatment will be computed. The treatment effects for the binary outcome (the incidence of PMPS) will be presented in terms of odds ratios and the associated 95% confidence intervals.

### Interim analyses {21b}

We do not anticipate significant safety issues associated with our study, as ketamine is an FDA-approved treatment. However, safety and efficacy will be monitored, with stopping rules developed in collaboration with the Data Safety and Monitoring Board (DSMB). We propose stopping rules for futility. If the stopping boundaries are crossed, the DSMB will consider recommending early stoppage of the trial after evaluating the totality of the data (including other endpoints such as death). We propose that the study not be stopped for early indications of efficacy because of the desire to develop models for personalized medicine, which requires a broad distribution of participant characteristics. A nonbinding futility boundary is proposed using O’Brien-Fleming-type beta-spending function at 50% information. According to this rule, the one-sided Z-score cutoff for Wilcoxon’s rank-sum test comparing each active arm to the placebo is 0.755. The DSMB will have discretion to recommend stopping the trial early if safety concerns become substantial.

### Methods for additional analyses (e.g., subgroup analyses) {20b}

Although RCTs provide information on the average treatment effects for the trial target population, these estimated treatment effects may vary considerably across patients, depending on the patients’ clinical, demographic, and health behaviors prior to randomization. We will conduct the following additional analyses to explore such heterogeneous treatment effects and to develop models for precision medicine: (1) subgroup analysis, (2) likely responder analysis, and (3) individualized treatment rules development.

1. We will conduct subgroup analysis to explore the treatment heterogeneity with respect to the following factors: (i) Participant-related factors: age, race, ethnicity, and prior narcotic use; (ii) types of elective breast surgery for oncologic indication as follows: unilateral or bilateral mastectomy, + /– lymph node dissection, reconstruction vs. none, and prophylactic vs. treatment mastectomy; (iii) other treatment: immunotherapy, hormonal therapy, chemotherapy and radiation therapy, and type of anesthesia techniques; (iv) baseline pain and other functional outcomes: BPI, PROMIS anxiety, and PROMIS depression short forms 4a, Pain Catastophizing Scale (PCS), PROMIS sleep disturbance, and PROMIS fatigue. For each factor, we will quantify moderation effects via the statistical tests for interactions between the treatment indicators and the candidate moderator in regression models for primary and secondary endpoints.
2. We will conduct likely responder analysis to study the treatment effects among likely responders (as opposed to among the whole population). We will investigate perioperative predictors (including clinical and psychosocial measures) of symptom response to ketamine, to search for classifiers constructed based on machine learning methods such as random forests that predict the individual-specific probabilities of developing PMPS at 3 months under treatment with continuous ketamine infusion and with single-dose ketamine [74]. By doing this, we can identify likely responders for each treatment and make statistical statements about whether the effects of the treatment among participants whose baseline characteristics fall in the classifier’s “treatment responder region” are causal with respect to the control treatment [75].
3. Based on the results from subgroup analyses, we will develop predictive models that predict treatment-specific outcomes based on pre-treatment patient characteristics (treatment effect moderators). These models will optimally combine treatment effect moderators to create a patient-specific treatment benefit index, which can be used to classify future patients into different treatment benefit strata (e.g., high benefit, low benefit, no benefit expected), based on pretreatment patient profiles. The development of such a score for optimal use of ketamine falls under the rubric of developing individualized treatment rules (ITRs) for optimizing clinical outcomes for future patients [76–78]. The goal of this ITR development is to guide ketamine treatment decisions by providing an estimate of the difference between treatment outcomes comparing the following: (1) single dose vs. placebo, (2) continuous infusion vs. placebo, and (3) single-dose vs. continuous infusion, for each individual patient, using a treatment benefit index [79]. A larger differential in favor of a particular treatment (i.e., a larger score) would indicate a more compelling reason for recommending use of that treatment to a given patient. We will balance two competing objectives when we develop ITRs: simplicity in terms of patient characteristics for practical implementation and accuracy in terms of predicting the treatment benefit of individual patients. We will also consider other approaches to developing ITRs, including regression trees, Q-learning, A-learning, and the outcome-weighted learning [77, 78, 80, 81]. The final optimal ITR will be selected based on consideration for the interpretability (for clinical implementation) and the ITR prediction performance (assessed via extensive internal cross-validation).

### Methods in analysis to handle protocol non-adherence and any statistical methods to handle missing data {20c}

Great effort will be made to prevent and avoid missing data within the limitations of a large multicenter trial. If the amount of missing data on the primary outcome is small (< 5%) and if it is determined that the data are missing completely at random (MCAR), then complete-case analyses will be used. However, if the amount of missing data is > 5% for the primary outcome, or if it is suspected that the missingness for the outcome measures does not follow MCAR upon detailed examination of reasons for missingness, then we will conduct a series of missing data analyses. First, we will assess the mechanism of missing data by comparing participants with and without missing values on baseline and other complete information, to detect any patterns in demographics or other characteristics associated with missing data. We will use multiple imputation, which imputes multiple values for each missing element to properly account for variability and provide correct inference [82]. From each multiply-imputed complete data, we will obtain estimates and standard errors using the same analytic methods for corresponding primary and secondary outcomes, and then combine final results using Rubin’s method [83]. Then we will use pattern mixture modeling to conduct sensitivity analyses under the missing not at random assumption and examine if the statistical findings are robust across several scenarios, including the least-favorable scenario where the missing data from the treatment arms follows the same pattern as that of the observed data from the placebo arm [84].

### Plans to give access to the full protocol, participant level-data, and statistical code {31c}

De-identified data will be made publicly available in accordance with the NIH HEAL Initiative Public Access and Data Sharing Policy.

## Oversight and monitoring

### Composition of the coordinating center and trial steering committee {5d}

The KALPAS study is part of the Pain Management Effectiveness Research Network, funded through the NIH Helping to End Addiction Long-term (HEAL) Initiative. The study is overseen by the NYU Grossman School of Medicine study team and the Trial Innovation Network (TIN). The TIN consists of Trial Innovation Centers (TICs) at Duke/Vanderbilt, Johns Hopkins University (JHU)/Tufts, University of Utah and the Recruitment Innovation Center (RIC) at Vanderbilt University Medical Center (VUMC). The Duke Clinical Research Institute, part of the Duke/Vanderbilt TIC, is the clinical coordinating center and provides site management and monitoring. The University of Utah is the data coordinating center (DCC) responsible for data management. The JHU/Tufts TIC is the statistical coordinating center, responsible for biostatistics and safety monitoring.

### Composition of the data monitoring committee, its role, and reporting structure {21a}

The study is overseen by an independent DSMB whose members are experts in anesthesiology, breast cancer, and statistics as well as a lay member who has expertise in breast cancer symptom and care management. The DSMB will meet approximately 6 months after start of enrollment and every 6 months afterwards. The DSMB will review study performance, monitor accrual of study participants, and track safety of study participants. The DSMB reports to the National Cancer Institute (NCI) on the safety and progress of the study and will provide recommendations on proceeding with the study, proceeding with modifications, or terminating the study.

### Adverse event reporting and harms {22}

Adverse events (AEs) will be recorded beginning at randomization and through postoperative day 7. All AEs will be assessed for severity, expectedness, and relatedness to study intervention. AEs will be submitted to the DCC within 7 business days of the study site investigator becoming aware of the event. All serious AEs will be reported within 24 h of study site investigator becoming aware of the event. All AEs will be followed until satisfactory resolution or until the study site physician deems the event to be chronic, secondary to oncologic diagnosis, and/or the participant is stable.

### Frequency and plans for auditing trial conduct {23}

Study staff will permit authorized representatives of the data and clinical coordinating centers (DCC & CCC), upon request, to review study records for source verification of study documentation, quality assurance reviews, audits, and evaluation of the study safety, progress, and data validity.

Quality control (QC) procedures will be implemented beginning with the data entry system, and data QC checks will be run on the database. Missing data or data anomalies will be communicated to the site(s) for clarification/resolution.

Following written SOPs, the monitors will verify that the clinical trial is conducted and data are generated, documented (recorded), and reported in compliance with the protocol, GCP, and the applicable regulatory requirements (e.g., good laboratory practice [GLP], good manufacturing practice [GMP]).

The investigational site will provide direct access to all trial-related sites, source data/documents, and reports for the purpose of monitoring and auditing by the sponsor and inspection by local and regulatory authorities.

### Plans for communicating important protocol amendments to relevant parties (e.g. trial participants, ethical committees) {25}

Any protocol modifications will be reviewed and approved by the University of Utah single IRB.

## Dissemination plans {31a}

The results of the study will be published in peer-reviewed journals.

## Discussion

This study will establish the effectiveness of two interventions, an established ketamine infusion regimen, and an innovative single-dose regimen, to reduce PMPS in women undergoing mastectomy for oncologic indication or risk reduction. Both regimens are easy to implement, with low costs and well-demonstrated safety profiles that can be readily scaled to standard clinical care. These therapies work independent of peripheral pathology and hence can be generalized to other CPSP syndromes. Our study also addresses a health disparity within the current opioid epidemic, as PMPS affects a predominantly female patient population with a growing opioid use rate, whose pain has previously been undertreated and understudied. The study sites cover diverse demographics, making the results highly generalizable.

## Trial status

Recruitment began January 2022. The current approved protocol is version 4, approved June 29, 2023. Recruitment is expected to be completed by October 2025.

### Supplementary Information

**Additional file 1.** SPIRIT checklist.

## Data Availability

De-identified data for underlying primary data will be made publicly available via the NIH HEAL Initiative central data repository. Policies for access to the database will be in accordance with the NIH HEAL Initiative Public Access and Data Sharing Policy.

## Abbreviations

CPSP: Chronic postsurgical pain
PMPS: Postmastectomy pain syndrome
KALPAS: Ketamine analgesia for long-lasting pain relief after surgery
NIH: National Institutes of Health
HEAL: Helping to End Addiction Long-term
NCI: National Cancer Institute
TIN: Trial Innovation Network
RCT: Randomized controlled trial
PACU: Postanesthesia care unit
BPI: Brief Pain Inventory
ICH: International Council for Harmonisation
GCP: Good clinical practice
AMS: Altered mental status
BMI: Body mass index
BCPQ: Breast Cancer Pain Questionnaire
PROMIS: Pain-Reported Outcomes Measurement Information System
PGIC: Patient Global Impressions of Change
PHQ: Patient Health Questionnaire
POD: Postoperative day
TAPS: Tobacco, alcohol, prescription medication, and other substances tool
PCS: Pain Catastrophizing Scale
GAD: Generalized anxiety disorder
AE: Adverse event
SAE: Severe adverse event
FWER: Familywise error rate
DCC: Data coordinating center
CCC: Clinical coordinating center
FDA: Food and Drug Administration
IRB: Institutional Review Board
CRF: Case report form
EDC: Electronic data apture
HIPAA: Health Insurance Portability and Accountability Act
NYU: New York University
HHS: Health and human services
CoC: Certificate of Confidentiality
PI: Principal investigator
mITT: Modified intention-to-treat
SD: Standard deviation
BCa: Bias-corrected and accelerated
MMRM: Mixed-model repeated measures
REML: Restricted maximum likelihood
DSMB: Data Safety and Monitoring Board
ITR: Individualized treatment rules
MCAR: Missing completely at random
JHU: John Hopkins University
RIC: Recruitment Innovation Center
VUMC: Vanderbilt University Medical Center
DMC: Data Monitoring Committee
QC: Quality control
SOP: Standard operating procedure
GLP: Good laboratory practice
GMP: Good manufacturing practice

## References

[1] Meretoja TJ, Leidenius MHK, Tasmuth T, Sipila R, Kalso E (2014). Pain at 12 months after surgery for breast cancer. JAMA.

[2] Andersen KG, Duriaud HM, Jensen HE, Kroman N, Kehlet H (2015). Predictive factors for the development of persistent pain after breast cancer surgery. Pain.

[3] Okamoto A, Yamasaki M, Yokota I, Mori M, Matsuda M, Yamaguchi Y (2018). Classification of acute pain trajectory after breast cancer surgery identifies patients at risk for persistent pain: a prospective observational study. J Pain Res.

[4] Dereu D, Savoldelli GL, Combescure C, Mathivon S, Rehberg B (2018). Development of a simple preoperative risk score for persistent pain after breast cancer surgery: a prospective observational cohort study. Clin J Pain.

[5] Wang L, Guyatt GH, Kennedy SA, Romerosa B, Kwon HY, Kaushal A (2016). Predictors of persistent pain after breast cancer surgery: a systematic review and meta-analysis of observational studies. CMAJ.

[6] Andersen KG, Kehlet H (2011). Persistent pain after breast cancer treatment: a critical review of risk factors and strategies for prevention. J Pain.

[7] Bruce J, Thornton AJ, Powell R, Johnston M, Wells M, Heys SD (2014). Psychological, surgical, and sociodemographic predictors of pain outcomes after breast cancer surgery: a population-based cohort study. Pain.

[8] Miaskowski C, Cooper B, Paul SM, West C, Langford D, Levine JD (2012). Identification of patient subgroups and risk factors for persistent breast pain following breast cancer surgery. J Pain.

[9] Tasmuth T, Blomqvist C, Kalso E (1999). Chronic post-treatment symptoms in patients with breast cancer operated in different surgical units. Eur J Surg Oncol.

[10] Jung BF, Ahrendt GM, Oaklander AL, Dworkin RH (2003). Neuropathic pain following breast cancer surgery: proposed classification and research update. Pain.

[11] Montes A, Roca G, Sabate S, Lao JI, Navarro A, Cantillo J (2015). Genetic and clinical factors associated with chronic postsurgical pain after hernia repair, hysterectomy, and thoracotomy: a two-year multicenter cohort study. Anesthesiology.

[12] Gilron I, Vandenkerkhof E, Katz J, Kehlet H, Carley M (2017). Evaluating the association between acute and chronic pain after surgery: impact of pain measurement methods. Clin J Pain.

[13] Schug SA, Bruce J (2017). Risk stratification for the development of chronic postsurgical pain. Pain Rep.

[14] Houle TT, Miller S, Lang JE, Booth JL, Curry RS, Harris L (2017). Day-to-day experience in resolution of pain after surgery. Pain.

[15] Domino EF (2010). Taming the ketamine tiger. 1965. Anesthesiology.

[16] Sadove MS, Shulman M, Hatano S, Fevold N (1971). Analgesic effects of ketamine administered in subdissociative doses. Anesth Analg.

[17] Sinner B, Graf BM (2008). Ketamine. Handb Exp Pharmacol.

[18] Sveticic G, Eichenberger U, Curatolo M (2005). Safety of mixture of morphine with ketamine for postoperative patient-controlled analgesia: an audit with 1026 patients. Acta Anaesthesiol Scand.

[19] Zhou H, Zhang Q, Martinez E, Dale J, Hu S, Zhang E (2018). Ketamine reduces aversion in rodent pain models by suppressing hyperactivity of the anterior cingulate cortex. Nat Commun.

[20] Wang J, Goffer Y, Xu D, Tukey DS, Shamir DB, Eberle SE (2011). A single subanesthetic dose of ketamine relieves depression-like behaviors induced by neuropathic pain in rats. Anesthesiology.

[21] Zhang Q, Manders T, Tong AP, Yang R, Garg A, Martinez E, et al. Chronic pain induces generalized enhancement of aversion. eLife. 2017;6. 10.7554/eLife.25302. 10.7554/eLife.25302 PMC5438248 28524819

[22] Zarate CA, Singh JB, Carlson PJ, Brutsche NE, Ameli R, Luckenbaugh DA (2006). A randomized trial of an N-methyl-D-aspartate antagonist in treatment-resistant major depression. Arch Gen Psychiatry.

[23] Berman RM, Cappiello A, Anand A, Oren DA, Heninger GR, Charney DS (2000). Antidepressant effects of ketamine in depressed patients. Biol Psychiatry.

[24] Ibrahim L, Diazgranados N, Franco-Chaves J, Brutsche N, Henter ID, Kronstein P (2012). Course of improvement in depressive symptoms to a single intravenous infusion of ketamine vs add-on riluzole: results from a 4-week, double-blind, placebo-controlled study. Neuropsychopharmacology.

[25] Diazgranados N, Ibrahim L, Brutsche NE, Newberg A, Kronstein P, Khalife S (2010). A randomized add-on trial of an N-methyl-D-aspartate antagonist in treatment-resistant bipolar depression. Arch Gen Psychiatry.

[26] Verret M, Lauzier F, Zarychanski R, Perron C, Savard X, Pinard AM (2020). Perioperative use of gabapentinoids for the management of postoperative acute pain: a systematic review and meta-analysis. Anesthesiology.

[27] Remerand F, Le Tendre C, Baud A, Couvret C, Pourrat X, Favard L (2009). The early and delayed analgesic effects of ketamine after total hip arthroplasty: a prospective, randomized, controlled, double-blind study. Anesth Analg.

[28] Yamauchi M, Asano M, Watanabe M, Iwasaki S, Furuse S, Namiki A (2008). Continuous low-dose ketamine improves the analgesic effects of fentanyl patient-controlled analgesia after cervical spine surgery. Anesth Analg.

[29] Adam F, Chauvin M, Du Manoir B, Langlois M, Sessler DI, Fletcher D (2005). Small-dose ketamine infusion improves postoperative analgesia and rehabilitation after total knee arthroplasty. Anesth Analg.

[30] Aveline C, Gautier JF, Vautier P, Cognet F, Hetet HL, Attali JY (2009). Postoperative analgesia and early rehabilitation after total knee replacement: a comparison of continuous low-dose intravenous ketamine versus nefopam. Eur J Pain.

[31] Kim SH, Kim SI, Ok SY, Park SY, Kim MG, Lee SJ (2013). Opioid sparing effect of low dose ketamine in patients with intravenous patient-controlled analgesia using fentanyl after lumbar spinal fusion surgery. Korean J Anesthesiol.

[32] Zakine J, Samarcq D, Lorne E, Moubarak M, Montravers P, Beloucif S (2008). Postoperative ketamine administration decreases morphine consumption in major abdominal surgery: a prospective, randomized, double-blind, controlled study. Anesth Analg.

[33] Webb AR, Skinner BS, Leong S, Kolawole H, Crofts T, Taverner M (2007). The addition of a small-dose ketamine infusion to tramadol for postoperative analgesia: a double-blinded, placebo-controlled, randomized trial after abdominal surgery. Anesth Analg.

[34] Garg N, Panda NB, Gandhi KA, Bhagat H, Batra YK, Grover VK (2016). Comparison of Small dose ketamine and dexmedetomidine infusion for postoperative analgesia in spine surgery–a prospective randomized double-blind placebo controlled study. J Neurosurg Anesthesiol.

[35] Wang L, Johnston B, Kaushal A, Cheng D, Zhu F, Martin J (2016). Ketamine added to morphine or hydromorphone patient-controlled analgesia for acute postoperative pain in adults: a systematic review and meta-analysis of randomized trials. Can J Anaesth.

[36] Assouline B, Tramer MR, Kreienbuhl L, Elia N (2016). Benefit and harm of adding ketamine to an opioid in a patient-controlled analgesia device for the control of postoperative pain: systematic review and meta-analyses of randomized controlled trials with trial sequential analyses. Pain.

[37] Barreveld AM, Correll DJ, Liu X, Max B, McGowan JA, Shovel L (2013). Ketamine decreases postoperative pain scores in patients taking opioids for chronic pain: results of a prospective, randomized, double-blind study. Pain Med.

[38] Nielsen RV, Fomsgaard JS, Siegel H, Martusevicius R, Nikolajsen L, Dahl JB (2017). Intraoperative ketamine reduces immediate postoperative opioid consumption after spinal fusion surgery in chronic pain patients with opioid dependency: a randomized, blinded trial. Pain.

[39] Loftus RW, Yeager MP, Clark JA, Brown JR, Abdu WA, Sengupta DK (2010). Intraoperative ketamine reduces perioperative opiate consumption in opiate-dependent patients with chronic back pain undergoing back surgery. Anesthesiology.

[40] Sing DC, Barry JJ, Cheah JW, Vail TP, Hansen EN (2016). Long-acting opioid use independently predicts perioperative complication in total joint arthroplasty. J Arthroplasty.

[41] Cron DC, Englesbe MJ, Bolton CJ, Joseph MT, Carrier KL, Moser SE (2017). Preoperative opioid use is independently associated with increased costs and worse outcomes after major abdominal surgery. Ann Surg.

[42] Waljee JF, Cron DC, Steiger RM, Zhong L, Englesbe MJ, Brummett CM (2017). Effect of preoperative opioid exposure on healthcare utilization and expenditures following elective abdominal surgery. Ann Surg.

[43] Faour M, Anderson JT, Haas AR, Percy R, Woods ST, Ahn UM (2017). Neck pain, preoperative opioids, and functionality after cervical fusion. Orthopedics.

[44] Lentine KL, Lam NN, Xiao H, Tuttle-Newhall JE, Axelrod D, Brennan DC (2015). Associations of pre-transplant prescription narcotic use with clinical complications after kidney transplantation. Am J Nephrol.

[45] Wilson JL, Poulin PA, Sikorski R, Nathan HJ, Taljaard M, Smyth C (2015). Opioid use among same-day surgery patients: prevalence, management and outcomes. Pain Res Manag.

[46] Jouguelet-Lacoste J, La Colla L, Schilling D, Chelly JE (2015). The use of intravenous infusion or single dose of low-dose ketamine for postoperative analgesia: a review of the current literature. Pain medicine.

[47] Boenigk K, Echevarria GC, Nisimov E, von Bergen Granell AE, Cuff GE, Wang J (2019). Low-dose ketamine infusion reduces postoperative hydromorphone requirements in opioid-tolerant patients following spinal fusion: a randomised controlled trial. Eur J Anaesthesiol.

[48] Laskowski K, Stirling A, McKay WP, Lim HJ (2011). A systematic review of intravenous ketamine for postoperative analgesia. Can J of anaesth.

[49] Doan LV, Wang J (2018). An update on the basic and clinical science of ketamine analgesia. Clin J Pain.

[50] Hayes C, Armstrong-Brown A, Burstal R (2004). Perioperative intravenous ketamine infusion for the prevention of persistent post-amputation pain: a randomized, controlled trial. Anaesth Intensive Care.

[51] Suzuki M, Haraguti S, Sugimoto K, Kikutani T, Shimada Y, Sakamoto A (2006). Low-dose intravenous ketamine potentiates epidural analgesia after thoracotomy. Anesthesiology.

[52] Mcnicol ED, Schumann R, Haroutounian S (2014). A systematic review and meta-analysis of ketamine for the prevention of persistent post-surgical pain. Acta Anaesthesiol Scand.

[53] Autry AE, Adachi M, Nosyreva E, Na ES, Los MF, Cheng PF (2011). Nmda receptor blockade at rest triggers rapid behavioural antidepressant responses. Nature.

[54] Garcia LS, Comim CM, Valvassori SS, Reus GZ, Barbosa LM, Andreazza AC (2008). Acute administration of ketamine induces antidepressant-like effects in the forced swimming test and increases bdnf levels in the rat hippocampus. Prog Neuropsychopharmacol Biol Psychiatry.

[55] Maeng S, Zarate CA, Du J, Schloesser RJ, McCammon J, Chen G (2008). Cellular mechanisms underlying the antidepressant effects of ketamine: role of alpha-amino-3-hydroxy-5-methylisoxazole-4-propionic acid receptors. Biol Psychiatry.

[56] Gould TD, O'Donnell KC, Dow ER, Du J, Chen G, Manji HK (2008). Involvement of Ampa receptors in the antidepressant-like effects of lithium in the mouse tail suspension test and forced swim test. Neuropharmacology.

[57] Koike H, Chaki S (2014). Requirement of Ampa receptor stimulation for the sustained antidepressant activity of ketamine and ly341495 during the forced swim test in rats. Behav Brain Res.

[58] Chourbaji S, Vogt MA, Fumagalli F, Sohr R, Frasca A, Brandwein C (2008). Ampa receptor subunit 1 (Glur-a) knockout mice model the glutamate hypothesis of depression. FASEB J.

[59] Yuen EY, Wei J, Liu W, Zhong P, Li X, Yan Z (2012). Repeated stress causes cognitive impairment by suppressing glutamate receptor expression and function in prefrontal cortex. Neuron.

[60] Liu H, Wen LM, Qiao H, An SC (2013). Modulation of hippocampal glutamate and Nmda/Ampa receptor by homocysteine in chronic unpredictable mild stress-induced rat depression. Sheng Li Xue Bao.

[61] Li N, Lee B, Liu RJ, Banasr M, Dwyer JM, Iwata M (2010). Mtor-dependent synapse formation underlies the rapid antidepressant effects of Nmda antagonists. Science.

[62] Zhou W, Wang N, Yang C, Li XM, Zhou ZQ, Yang JJ (2014). Ketamine-induced antidepressant effects are associated with ampa receptors-mediated upregulation of mtor and bdnf in rat hippocampus and prefrontal cortex. Eur Psychiatry.

[63] Yang C, Hu YM, Zhou ZQ, Zhang GF, Yang JJ (2013). Acute administration of ketamine in rats increases hippocampal Bdnf and Mtor levels during forced swimming test. Ups J Med Sci.

[64] Dale J, Zhou H, Zhang Q, Martinez E, Hu S, Liu K (2018). Scaling up cortical control inhibits pain. Cell Rep.

[65] Sheikh S, Hendry P (2018). The expanding role of ketamine in the emergency department. Drugs.

[66] Karlow N, Schlaepfer CH, Stoll CRT, Doering M, Carpenter CR, Colditz GA (2018). A systematic review and meta-analysis of ketamine as an alternative to opioids for acute pain in the emergency department. Acad Emerg Med.

[67] Pourmand A, Mazer-Amirshahi M, Royall C, Alhawas R, Shesser R (2017). Low dose ketamine use in the emergency department, a new direction in pain management. Am J Emerg Med.

[68] Wang J, Echevarria GC, Doan L, Ekasumara N, Calvino S, Chae F (2019). Effects of a single subanaesthetic dose of ketamine on pain and mood after laparoscopic bariatric surgery: a randomised double-blind placebo controlled study. Eur J Anaesthesiol.

[69] Cleeland CS, Ryan KM (1994). Pain assessment: global use of the Brief Pain Inventory. Ann Acad Med Singapore.

[70] Dworkin RH, Turk DC, Farrar JT, Haythornthwaite JA, Jensen MP, Katz NP (2005). Core outcome measures for chronic pain clinical trials: immpact recommendations. Pain.

[71] Kean J, Monahan PO, Kroenke K, Wu J, Yu Z, Stump TE (2016). Comparative responsiveness of the PROMIS pain interference short forms, Brief Pain Inventory, Peg, and Sf-36 Bodily Pain Subscale. Medical Care.

[72] Kroenke K, Theobald D, Wu J, Tu W, Krebs EE (2012). Comparative responsiveness of pain measures in cancer patients. J Pain.

[73] Piaggio G, Elbourne DR, Pocock SJ, Evans SJ, Altman DG, Group C (2012). Reporting of noninferiority and equivalence randomized trials: extension of the CONSORT 2010 statement. JAMA.

[74] Breiman L (2001). Random forests. Mach Learn.

[75] Laska E, Siegel C, Lin Z (2022). A likely responder approach for the analysis of randomized controlled trials. Contemp Clin Trials.

[76] Steyerberg EW, Steyerberg EW (2009). Study design for prediction models. Clinical Prediction Models: A Practical Approach to Development, Validation, and Updating.

[77] Qian M, Murphy SA (2011). Performance guarantees for individualized treatment rules. Ann Stat.

[78] Zhao Y, Zeng D, Rush AJ, Kosorok MR (2012). Estimating individualized treatment rules using outcome weighted learning. J Am Stat Assoc.

[79] Park H, Tarpey T, Liu M, Goldfeld K, Wu Y, Wu D (2022). Development and validation of a treatment benefit index to identify hospitalized patients with Covid-19 who may benefit from convalescent plasma. JAMA Netw Open.

[80] Kosorok MR, Laber EB (2019). Precision medicine. Annu Rev Stat Appl.

[81] Hahn PR, Murray JS, Carvalho CM (2020). Bayesian regression tree models for causal inference: regularization, confounding, and heterogeneous effects (with discussion). Bayesian Anal.

[82] Azur MJ, Stuart EA, Frangakis C, Leaf PJ (2011). Multiple imputation by chained equations: what is it and how does it work?. Int J Methods Psychiatr Res.

[83] Toutenburg H, Rubin DB (1990). Multiple imputation for nonresponse in surveys. Stat Pap.

[84] Little RJ, Wang Y (1996). Pattern-mixture models for multivariate incomplete data with covariates. Biometrics.

